# Persistence of goitre in the post-iodization phase: micronutrient deficiency or thyroid autoimmunity?

**Published:** 2011-01

**Authors:** Sambit Das, Anil Bhansali, Pinaki Dutta, Arun Aggarwal, M.P. Bansal, Dinesh Garg, Muthuswamy Ravikiran, Rama Walia, Vimal Upreti, Santosh Ramakrishnan, Naresh Sachdeva, Sanjay K. Bhadada

**Affiliations:** *Department of Endocrinology, Postgraduate Institute of Medical Education & Research, Chandigarh, India*; * *Department of Community Medicine, Postgraduate Institute of Medical Education & Research, Chandigarh, India*; ** *Department of Biophysics, Panjab University, Chandigarh, India*; + *IDD Project, Chandigarh, India*

**Keywords:** Goitre, iodine deficiency, iron deficiency, selenium, thyroid autoimmunity

## Abstract

**Background & objectives:**

Despite years of salt iodization, goitre continues to be a major public health problem worldwide. We examined the prevalence of goitre in the post-iodization phase and the relationship of goitre with micronutrient status and thyroid autoimmunity in school children of Chandigarh, north India.

**Methods:**

Two phase study; in the first phase, 2148 children of 6 to 16 yr were screened for goitre by two independent observers as per the WHO grading system. In the second phase, a case-control study, 191 children with goitre and 165 children without goitre were compared with respect to urinary iodine, iodine content of salt, serum levels of T_3_, T_4_, TSH, anti-TPO (thyroid peroxidase) antibody, haemoglobin, ferritin and selenium.

**Results:**

Prevalence of goitre in the studied subjects was 15.1 per cent (13.9% in 6 to 12 yr and 17.7% in 13 to 16 yr age group, *P*= 0.03). Median urinary iodine excretion in both the groups was sufficient and comparable (137 and 130 µg/l). 3.2 per cent children with goitre and 2.4 per cent without goitre had hypothyroidism (subclinical and clinical) and only one child with goitre had subclinical hyperthyroidism. Nine (4.9%) children in the goitre group and 3 (1.9%) in control group had anti-TPO antibody positivity. The median serum selenium levels were not different in both the groups (181.9 and 193.5 µg/l). Seventy one (37.4%) of the goitrous children had anaemia (haemoglobin <12 g/dl) as compared to 41 (24.8%) of the control group (*P* <0.01). More number of goitrous children (39, 20.6%) were depleted of tissue iron stores (serum ferritin <12 µg/l) as compared to controls (11, 6.4%; *P*<0.001). Serum ferritin level negatively correlated with the presence of goitre (r = - 0.22, *P* =0.008) and had an OR of 2.8 (CI 1.20 - 6.37, *P* =0.017).

**Interpretation & conclusions:**

There was a high prevalence of goitre in young children despite iodine repletion and low thyroid autoimmunity. The concurrent iron deficiency correlated with the presence of goiter. However, the cause and effect relationship between iron deficiency state and goitre requires further elucidation.

Iodine is an essential micronutrient required for structural development and optimal functional activity of the thyroid gland and central nervous system. Iodine deficiency has been shown to be associated with endemic cretinism, endemic goitre and subcretinous mental subnormalities^1^. A study done in Kangra valley in 1973, a sub Himalayan iodine depleted region, showed that after 6 years of iodized salt supplementation, there was an appreciable decline in goitre prevalence from 40 to 15 per cent^2^. After this landmark observation and testimony to iodine deluge, the government of India in 1987 decided to implement universal iodization programme^3^.

Despite being iodine replete, goitre continues to be prevalent in mild to moderate degree of endemicity in most states of India^4^–^8^. These observations suggest that there might be other goitrogens or deficiency of other micronutrients responsible for the persistence of goitre despite adequate salt iodization. Autoimmune thyroid disorders are also an important cause of goitre in the post-iodization phase in India as shown earlier^5^. Several micronutrient deficiencies have been incriminated as the cause of goitre other than iodine; notable among these are iron and selenium deficiency. Iron deficiency is associated with high prevalence of goitre in Iranian school children^9^ and similar result was shown in sub-Saharan Africa^10^. Similarly selenium levels in serum and urine correlated with goitre prevalence in Germany^11^, Poland^12^ and Turkey^13^. Thiocyanate has also been implicated as a goitrogen, as shown in a previous study from India^5^.

The current study was aimed to survey the prevalence of goitre in the post-iodization phase and the relationship of goitre with micronutrient status and thyroid autoimmunity in school children of the Union Territory of Chandigarh, a city of northern India.

## Material & Methods

**Fig. 1:**
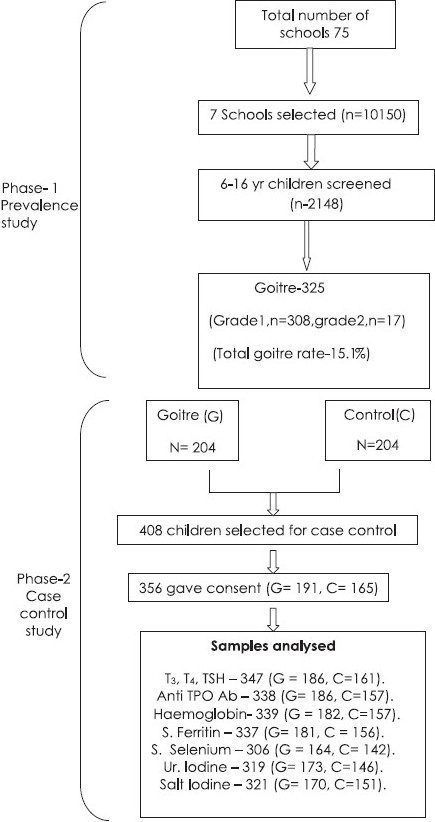
Outline of the study.

The study was conducted in two phases (Fig. 1). In the first phase (June 2007 to March 2008), list of all the schools (both government and private) was procured from UT school authority of Chandigarh and seven out of 75 schools were selected by systematic random sampling. A total of 2148 children (6-16 yr) selected by simple random sampling were screened for goitre. The second phase (April-July 2008) of the study was a case-control study in which children with goitre (taken as cases) were compared with age and sex matched controls (children without goitre) from the same cohort. Three levels of consent were taken: first one from the school authority of Chandigarh, second from principals of each school surveyed, and lastly from the parents of the students whose blood and urine samples were taken. The study protocol was approved by the institute ethics committee of Post- graduate Institute of Medical Education & Research, Chandigarh.

Goitre was screened by palpation method and was graded as per definition provided by WHO/UNICEF/ICCIDD^1^. The grading is described as Grade-0, no palpable or visible goiter; Grade-1, goitre that is palpable but not visible when the neck is in the normal position; and Grade-2, visible when the neck is in the normal position.

Goitre was examined by 2 trained physicians independently, and if the observation was not concordant, the lower grade recorded by either of them was taken as the grade of the goitre.

In the second phase of the study, 204 children with goitre and similar number (n=204) of children without goitre were selected from the same cohort for the subsequent case-control study. Consent could be obtained in 356 children (Goitre-191, Control-165). The demographic parameters, history and physical examination were recorded as per the protocol. The smoking habit of the parents was obtained through the proforma supplied to the children. The socio-economic status was assessed by modified Kuppuswamy scale^14^.

Spot urine samples were collected in a wide mouthed iodine free plastic tubes with tight screw tops and kept in a refrigerator at -4°C after addition of 1 drop of toluene. Urinary iodine was estimated by ammonium per sulphate method^1^. Salt samples were collected from the salt packets used at home in closed polythene bags and salt iodine content was determined by titrimetric method^1^.

Under aseptic condition, 7 ml of blood was collected from ante-cubital vein in both plain and EDTA vaccutainers. EDTA samples were processed for haemoglobin estimation within 6 h of collection of the samples. Blood samples collected in the plain vials were immediately centrifuged at -4°C, transported to the Endocrinology laboratory with proper maintenance of cold chain and were stored at -20°C. Haemoglobin was determined by colorimetric method^15^.

Serum T_3_(tri-iodothyronine), T_4_(tetraiodothyronine), TSH (thyroid stimulating hormone), anti-TPO (thyroid peroxidase) antibody and serum ferritin were determined by immunochemiluminiscence (ICMA Elecsys 2010, Roche, Germany). The normal range and coefficient of variation (CV) of various parameters are listed in Table I. Serum ferritin level <50 µg/l was considered as iron deficient state and <12 µg/l as severe iron deficient state^16^. Serum selenium was determined by fluorometric method^17^ and results were analysed in quartiles.

**Table I:** The normal range of various biochemical parameters

Biochemical parameter	Normal range	Coefficient of variation (%) (intra and interassay)
Serum T_3_ (ng/ml)	0.8-2	4.1-5.4
Serum T_4_ (µg/dl)	4.8-12.7	3.4-4.2
Serum TSH (µIU/ml)	0.27-4.2	0.27-4.2
Anti-TPO antibody (IU/ml)	<34	4.3-9.5
Serum ferritin (µg/l), Males	30-400	3.0-3.9
Serum ferritin (µg/l), Females	13-150	3.0-3.9

Depending on the thyroid function tests (TFT) results, children were classified into euthyroid (normal T_3_, T_4_and TSH), subclinical hypothyroid (TSH>97^th^ percentile of the group studied with normal T_4_and T_3_), hypothyroid (TSH>97^th^ percentile with T_4_< 4.8 µg/dl), subclinical hyperthyroid (TSH<0.27 µIU/ml with normal serum T_4_and T_3_) and hyperthyroid (TSH<0.27 µIU/ml with T_4_>12.7 µg/dl and T_3_>2 ng/ml)^18^. Anti-TPO antibody titre of ≥34 IU/ml was considered as positive for thyroid autoimmunity.

### 

#### Statistcial analysis:

The calculated sample size was 1400 assuming expected goitre prevalence to be 10 per cent; 122 goitrous children were planned to be compared with similar number of age and sex matched controls with 95 per cent confidence interval and 80 per cent power of the study. SPSS 13 (SPSS inc., Chicago) was used for statistical analysis. Number and percentage were calculated for categorical data. Fisher’s exact test and Chi-square test were applied to examine difference between proportions at a level of significance of 0.05. For continuous quantitative data mean, standard deviation and median were calculated. Data were checked for normal distribution with Kolmogorov-Smirnov test. For skewed data Mann-Whitney test was used to compare difference between means and for normally distributed data unpaired t test was applied. Multivariate regression analysis was done to predict the independent variables associated with goitre. Spearman’s correlation was used for assessing the correlation between goiter, haemoglobin and ferritin levels. Finally, the power of the study was 85 per cent with 95 per cent confidence interval.

## Results

**Fig. 2:**
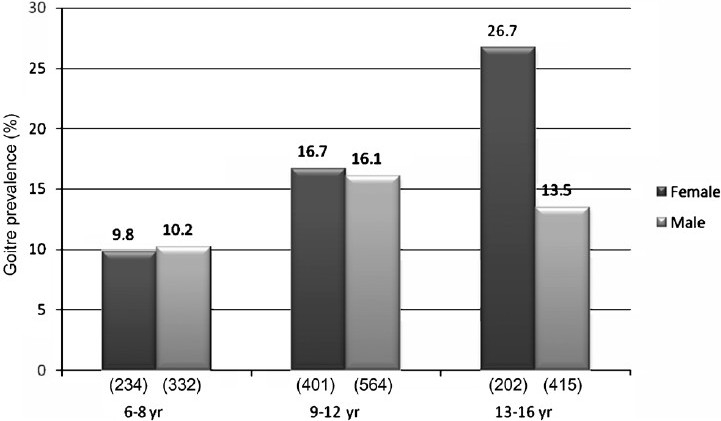
Goitre prevalence in different age and sex groups.

The prevalence of goitre in the studied population (n=2148) was 15.1 per cent (n=325). The prevalence of goiter was higher in the adolescents (13 to 16 yr) as compared to younger children (6 to 12 yr) (17.7 and 13.9%, *P*=0.03). All had diffuse goiter; 14.3 per cent (n=308) had grade-1 and 0.8 per cent (n=17) had grade-2 goitre. The inter and intra-observer variations in grading of goitre were 2.7 and 2 per cent respectively. Goitre was more prevalent in girls than in boys (17.2 vs 13.8%, *P*=0.05) and this sex difference was more marked in 13 to 16 yr of age group (*P*=0.01; Fig. 2.)

### 

#### Use and knowledge of iodized salt:

Parents of 82.4 per cent children knew that the salt they used in their houses contained iodine in it. Use of iodised salt was reported in families of 149 (84.2%) children with goitre and 126 (83.4%) control children in their day to day life. Only 155 (45.5%) of parents knew the advantages of iodized salt. Only 78 (23.5%) of parents knew that iodized salt prevents goitre.

#### Salt and urinary iodine:

The mean iodine content of salt in 321 samples was 36.47 ± 6.44 ppm. The iodine content of the salt samples from goitre and control groups was comparable (36.55 ± 6.28 vs 38.38 ± 6.63 ppm). Majority (315) of the samples (98.1%) had salt iodine content of >15 ppm. The median urinary iodine excretion in children with goitre and control group were 137 and 130 µg/l respectively. None of the children had urinary iodine excretion <20 µg/l.

#### Thyroid function status:

No differences in mean T_3_, T_4_ and TSH were found between goitre and control groups (T3 = 1.64 ± 0.30 vs 1.62 ± 0.27 ng/ml, T4=9.16 ± 1.9 vs 8.99 ± 1.91 µg/dl, TSH=3.92 ± 4.00 vs 3.39 ± 2.28 µIU/ml) Nine (2.6%) children had subclinical hypothyroidism, one (0.3%) had overt hypothyroidism, 1 (0.3%) child had subclinical hyperthyroidism and none had overt hyperthyroidism. Five (2.7%) children with goitre and 4 (2.4%) without goitre had subclinical hypothyroidism. One child with overt hypothyroidism and another with subclinical hyperthyroidism had goitre.

#### Thyroid autoimmunity:

A total of 12 (3.5%) children were anti-TPO antibody positive (≥ 34 IU/ml), 4.9 per cent in goitre group and 1.9 per cent in the control group. There was no sex predilection for anti-TPO antibody positivity (male 2.8%, female 4.4%) but more children with deranged thyroid hormonal status (subclinical hypothyroidism, overt hypothyroidism and subclinical hyperthyroidism) had anti-TPO antibody positivity than euthyroid children (9.5 vs 1.9%, *P*=0.005).

#### Anaemia and iron status:

Anaemia (Hb<12 g/dl) was present in (31.7%) of children. Haemoglobin was significantly lower in children with goitre than in the control group (12.0 ± 1.2 vs 12.4 ± 1.0 g/dl, *P*=0.01) and 37.4 per cent of goitrous children and 24.8 per cent of control children had haemoglobin levels <12 g/dl (*P*<0.05). There was a significant difference in ferritin levels among goitrous and control children (median values 22.2 and 28.3 µg/l, *P*=<0.001). Serum ferritin level was <50 µg/l in 86.7 per cent children in goitre group compared to 78.8 per cent in controls (*P*<0.057) and it was <12 µg/l (severe iron deficiency state) in 20.6 per cent of goitrous children as compared to 6.4 per cent of control children (*P*<0.001).

On further analysis, 35.2 per cent of children with goitre and 21.1 per cent without goitre in the age group of 6 to12 yr were anaemic (*P*<0.01) while 43.4 per cent (23/53) of adolescents (13-16 yr) with goitre and 34.9 per cent of those without goitre had anaemia. In girls of 13 to16 yr of age group, 44.4 per cent of cases with goitre had anaemia as compared to 50 per cent in the control group.

In the 6 to12 yr age group, 19.5 per cent of those with goitre had serum ferritin levels <12 µg/l as compared to 6.1 per cent in the control group (*P*<0.05). In the 13 to16 yr age group, 23.5 per cent of adolescents had serum ferritin <12 µg/l as compared to 7.3 per cent among controls (*P*<0.05). In girls of 13 to 16 yr age group, 29.6 per cent with goitre had serum ferritin <12 µg/l as compared to 13 per cent among controls. Thus, anaemia was more prevalent in adolescent girls in both the cases and control group.

#### Selenium status:

The median serum selenium value was not statistically different in the goitre and control group (191.0 vs 193.5 µg/l) and distribution of goitre in different quartiles of serum selenium was not statistically different.

#### Smoking habit:

17.3 per cent of goitrous children and 18.8 per cent of control children had history of passive smoking (*P*=0.82) and none were active smoker.

#### Socio-economic status and scholastic performance:

Socio-economic status was not different between the two groups. Maternal educational status (below class X^th^) in children with goitre was poorer as compared to the controls (70.7 vs 58.2%, *P*=0.01), but similar difference in paternal education was not found (50.8 vs 41.8%, *P*=0.09). Scholastic performance in terms of failure rate was higher in children with goitre than controls (14.1 and 7.6%, *P*= 0.08), and it did not correlate with thyroid function status, anaemia and selenium levels.

On multiple regression analysis considering goitre as a dependent variable and gender, urinary iodine, haemoglobin, serum ferritin, anti-TPO antibody positivity, serum selenium and smoking as independent variables, low serum ferritin had a significant OR of 2.8 (CI 1.20-6.37, *P*=0.017). The results for other variables did not achieve statistical significance (Table II). Using Spearman’s correlation, serum ferritin (r =-0.22, *P*=0.008) and Hb (r=-0.14, *P*=0.007) were negatively correlated with the presence of goitre.

**Table II:** Comparison of clinical and biochemical parameters in goitre and control groups

Parameters	Goitre group (n=191)	Control group (n=165)	*P* value	Odds ratio (95% CI)
Age (mean) in yr	11.0 ± 1.9	10.9 ± 2.21	0.4	-
Sex distribution (male: female)	112:79	79:86	-	-
Salt iodine (mean± SD) (ppm)	36.5±6.3	38.4±6.7	0.45	-
Median urinary iodine excretion (µg/l)	137	130	0.74	-
Hypothyroidism (subclinical and clinical) (%)	3.1	2.4	0.7	-
Anti TPO antibody positivity (%) (≥ 34 IU/ml)	4.9	1.9	0.22	6.1 (CI 0.72-52.8) *P*=0.098
Haemoglobin (mean± SD) (gm/dl)	12.0 ± 1.2	12.38 ± 1.06	0.01	-
Anaemia (haemoglobin <12 gm/dl) (%)	37.4	24.8	0.013	1.6 (CI 0.91-2.66) *P*=0.11
Iron deficiency (serum ferritin<12 µg/l) (%)	20.6	6.4	0.001	2.8 (CI 1.20-6.37) *P*=0.017
Serum selenium (median) µg/l	191.0	193.5	0.45	-
Smoking habit (active and passive) (%)	17.3	18.8	0.82	0.98 (CI 0.54-1.78) *P*=0.948

## Discussion

Our results showed that despite iodine sufficiency in this region, prevalence of goitre was 15 per cent. Selenium deficiency and thyroid autoimmunity did not contribute to the high prevalence of goitre, however, coexistent iron deficiency state correlated with the presence of goitre.

Rastogi *et al*^19^ reported the goitre prevalence of 16.4 per cent in Chandigarh. Subsequently in 1973 from a nearby rural area of Raipur Rani and hilly area of Morni, the goitre prevalence was found to be 15.5 and 38.8 per cent respectively^20^. In 2004, a study from Panchkula, a nearby area of Chandigarh, reported goitre prevalence of 12.9 per cent in children and 14.4 per cent in adolescents (unpublished observation).

Conventionally, in the goitre survey programmes, children in the age group of 6 to 12 yr are included to avoid the influence of hormonal alterations during peripubertal period on thyroid gland and possibly operational feasibility. In our study, children of 6 to 16 yr age group were included and on subhoc analysis the prevalence of goitre, as expected, was higher in the age group of 13 to 16 yr as compared to 6 to 12 yr. The prevalence of goitre in the age group of 6 to 12 yr in other studies varied between 7 to 37.6 per cent^4^,^6^,^7^,^21^ and in our study it was 13.9 per cent. Two studies which examined the prevalence of goitre between 6 to 18 yr and 6 to 16 yr age reported the prevalence to be 23.1 and 16.7 per cent respectively^5^,^8^, comparable to our data (15.1%). The observation of greater prevalence of goitre in the girls compared to the boys in the higher age groups was similar to that reported earlier^5^. This probably is related to the difference in sex hormones and pubertal growth pattern among boys and girls in higher age groups^5^,^22^.

In most of the studies goitre prevalence has been assessed by palpation method as suggested by WHO for community surveys^1^, however, ultrasonography of the thyroid would have been more objective to define the size of the gland. In our study, the inter- and intraobserver variations for goitre detection were 2.7 and 2 per cent respectively, which is well acceptable.

Our study population was iodine sufficient as evidenced by normal median urinary iodine excretion. Persistence of endemic goitre after adequate iodization have been observed by many authors all over the world, similar to our observation^23^,^24^. This suggests that there might be other goitrogens or factors playing a role besides iodine deficiency that need to be explored.

After iodine supplementation programme, thyroid autoimmunity has been considered as an important cause for persistence of goitre^23^,^25^. However, a study from India^5^found thyroid antibody positivity in only 7.3 per cent of children which is in concordance to our study. Several authors have assessed thyroid function status in goitrous children from both endemic and iodine supplemented regions and found the prevalence of hypothyroidism (subclinical and overt) ranging from 0-40 per cent^26^,^27^. A large study in India has found that 4.3 per cent children with goitre and 2.3 per cent of normal children had subclinical hypothyroidism^5^, a finding similar to our study.

Iron deficiency state is an important cause for persistence of goitre in the iodine replete population^9^,^10^. Hess *et al*^28^ have shown that supplementing iron in iron-deficient children with goitre decreases its size. In the present study, anaemia and iron deficiency as assessed by serum ferritin levels were significantly more prevalent in goitrous children than in controls in both the age groups (6 to 12 and 13 to 16 yr) and serum ferritin levels negatively correlated with the presence of goitre. As the study population included adolescents of 13 to 16 yr age group and iron deficiency is likely to be more prevalent in this age group particularly in female children, it would have influenced the prevalence of goitre. However, this attribute was counteracted by age and sex matched control group who also had iron deficiency of similar magnitude.

Selenium as a causative factor for goitre formation has been explained by diminished activity of selenocysteine enzymes in thyroid, notably glutathione peroxidase and deiodinase type-1 in selenium deficiency^29^,^30^. Though the normal range of serum selenium level has not been defined, plasma selenium concentration of 70-90 µg/l is required for optimal activity of glutathione peroxidase as shown in many experimental models^31^. Low selenium level has been shown in several areas of Iran, middle east and some European countries and it has been linked to the persistence of high goitre prevalence^13^,^31^. In the present study, there was no deficiency of selenium in the studied population. However, the goitrogenic effect of selenium deficiency is evident only when there is concurrent iodine deficiency^11^.

Contrary to the previous observations that smoking (both active and passive) is an important goitrogen^32^, there was no difference in the incidence of active and passive smoking between goitrous children and controls in our study. This could be explained by improved iodine status in our population inhibiting the goitrogenic effect of smoking, as was observed in a previous study^32^. In our study, there was no difference in socio-economic status between subjects with goitre and the control group, signifying absence of a role of socio-economic status in goitre prevalence in our population, a finding different from the previous studies^5^,^33^.

In conclusion, there was a high prevalence of goitre in young children in c0 handigarh, north India despite iodine sufficiency and low thyroid autoimmunity. The concurrent iron deficiency might be decreasing the effectiveness of iodine supplementation programme. The cause and effect relationship between iron deficiency and goitre requires further elucidation.
